# Distinct chromatin structures at the monoamine oxidase‐A promoter correlate with allele‐specific expression in SH‐SY5Y cells

**DOI:** 10.1111/gbb.12483

**Published:** 2018-05-11

**Authors:** M. Manca, V. Pessoa, P. Myers, A. Pickles, J. Hill, H. Sharp, C. Murgatroyd, V. J. Bubb, J. P. Quinn

**Affiliations:** ^1^ Department of Molecular and Clinical Pharmacology, Institute of Translational Medicine University of Liverpool Liverpool UK; ^2^ Institute of Psychology, Health and Society University of Liverpool Liverpool UK; ^3^ King's College London, MRC Social Genetic and Developmental Psychiatry Research Centre Institute of Psychiatry London UK; ^4^ School for Psychology and Clinical Language Sciences University of Reading Reading UK; ^5^ School of Healthcare Science Manchester Metropolitan University Manchester UK

**Keywords:** chromatin, epigenetics, gender, gene expression, MAOA, mental health, transcription, VNTR, X chromosome, X inactivation

## Abstract

Monoamine oxidase‐A (MAOA) metabolises monoamines and is implicated in the pathophysiology of psychiatric disorders. A polymorphic repetitive DNA domain, termed the uVNTR (upstream variable number tandem repeat), located at the promoter of the *MAOA* gene is a risk factor for many of these disorders. MAOA is on the X chromosome suggesting gender could play a role in regulation. We analysed MAOA regulation in the human female cell line, SH‐SY5Y, which is polymorphic for the uVNTR. This heterozygosity allowed us to correlate allele‐specific gene expression with allele‐specific transcription factor binding and epigenetic marks for MAOA. Gene regulation was analysed under basal conditions and in response to the mood stabiliser sodium valproate. Both alleles were transcriptionally active under basal growth conditions; however, the alleles showed distinct transcription factor binding and epigenetic marks at their respective promoters. Exposure of the cells to sodium valproate resulted in differential allelic expression which correlated with allele‐specific changes in distinct transcription factor binding and epigenetic marks at the region encompassing the uVNTR. Biochemically our model for MAOA promoter function has implications for gender differences in gene × environment responses in which the uVNTR has been implicated as a genetic risk.

## 1 INTRODUCTION

**Figure 1:**
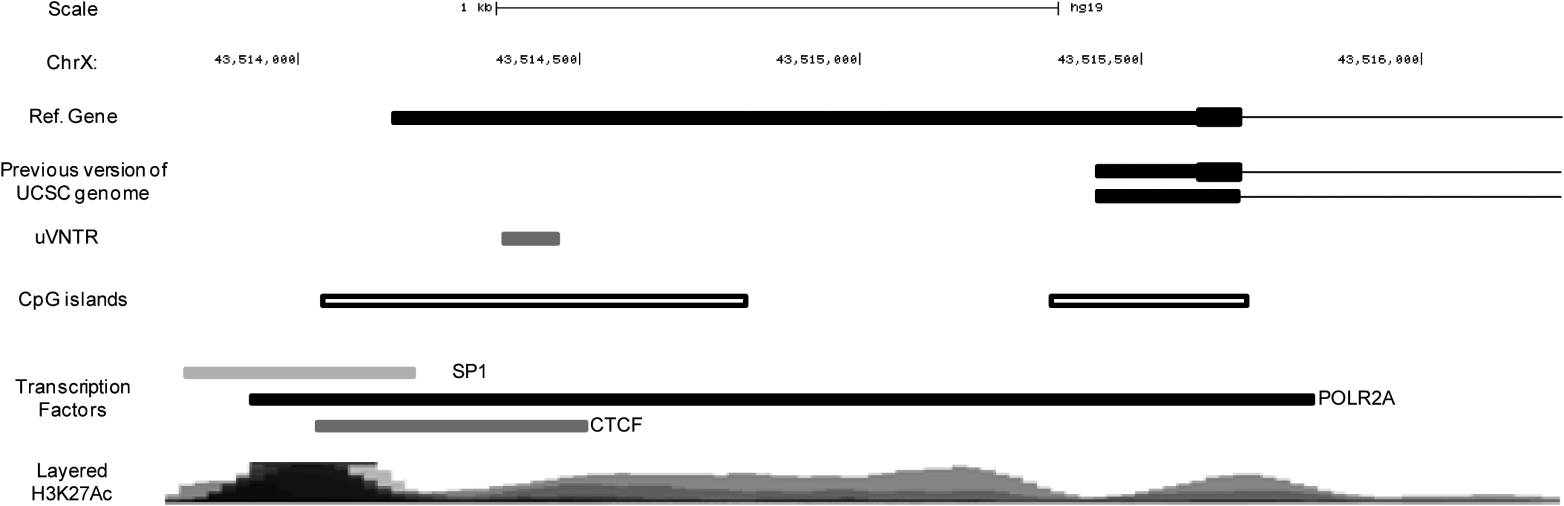
Graphic representation of MAOA promoter region based on The University of California Santa Cruz (UCSC) genome web browser, Hg38. From top to bottom: reference gene sequence; previous version of UCSC genome using Hg19; upstream tandem repeat: uVNTR; CpG islands; Encyclopedia of DNA Elements (ENCODE) transcription factor data (data version: ENCODE Mar 2012 Freeze) indicating those factors shown to bind to this region in specific cell lines, in short the darker the line the more confidence in the association; ENCODE data for H3K27Ac mark (often found near active regulatory elements) on 7 cell lines

Monoamine oxidase‐A (MAOA) metabolises monoamines and as such it is implicated in the pathophysiology of stress‐related illnesses, including major depressive disorder, addiction and violent behaviour.^1^, ^2^ A major mediator of MAOA expression is a polymorphic repetitive domain termed the uVNTR (upstream variable number tandem repeat) located in what was considered the promoter of the major transcript of the *MAOA* gene.^3^ The uVNTR is a 30 base pair repeat element that can be present in 2, 3, 3.5, 4 or 5 copies. The 2, 3 and 5 repeat variants are defined as low expression (MAOA‐L) alleles whilst the 3.5 and 4 repeat uVNTR are considered high expression variants (MAOA‐H).^4^ These low and high expression alleles can represent either a risk or a protective factor depending on the disorder studied.^4^, ^5^, ^6^, ^7^, ^8^, ^9^, ^10^, ^11^ It is thought that the uVNTR acts as a classical transcriptional regulator in the promoter of the *MAOA* gene and that the distinct variants support differential MAOA expression in a tissue‐specific and stimulus inducible manner. This gene and the uVNTR polymorphism are amongst the most often cited example of a genotype by environment interaction (G × E) in which an individual's genotype moderates the effect of environmental experience to alter mental health outcomes.^6^ Simplistically, the function of the uVNTR is modified by the complement of active transcription factors in the cell, altered in response to the environment, that are then able to recognise and bind to the uVNTR thus allowing the domain to act as a transcriptional regulator. However, bioinformatic data now indicates that there are at least 2 transcriptional start sites (TSSs) for the *MAOA* gene, a further isoform is produced from a more 5′ TSS. This newly described TSS now places the uVNTR in the 5′UTR (5′ untranslated region) of the *MAOA* gene, Figure 1, adding to the complexity of transcriptional regulation at this locus.

MAOA is located on the X chromosome and therefore genetic associations of the uVNTR with neuropsychiatric disorders in the male are simplified by having only one allele. However, associations of the uVNTR in females are complicated by not knowing the contribution of each allele to MAOA expression and the potential for one allele to exhibit X inactivation. This problem is made more difficult as the MAOA locus has been proposed as one of the loci that can escape X inactivation.^12^, ^13^, ^14^ To address the mechanisms which might underpin allele expression in the female we took advantage of the human neuroblastoma‐derived SH‐SY5Y cell line which has a female karyotype and is heterozygous for the uVNTR. This heterozygosity of the uVNTR allows us to distinguish allelic‐specific differences in epigenetic marks and transcription factor binding to the DNA encompassing the uVNTR in addition to identification of allele‐specific expression directed by the most 5′ TSS. In this communication we were able to show that both alleles, at least in the population of SH‐SY5Y cells, can support mRNA expression. This phenomenon was also observed in cDNA prepared from saliva from females who were heterozygous for the uVNTR. Using chromatin immunoprecipitation (ChIP) analysis in SH‐SY5Y cells we show that each allele can support distinct transcription factor, histone‐binding and methylation patterns. The pattern of factor binding and methylation over each allele was also correlated with a switch in allele‐specific gene expression in response to sodium valproate in SH‐SY5Y cells.

## 2 MATERIALS AND METHODS

### 2.1 Cell culture

The human neuroblastoma cell line SH‐SY5Y (ATCC/CRL‐2266) which is near diploid (47 chromosomes)^15^ was cultured in a 1:1 mix of Dulbecco's EMEM (Sigma, Dorset, UK) and Ham's F12 (Sigma) media supplemented with 10% (vol/vol) foetal bovine serum, 2 mM L‐glutamine (Sigma), 1 mM sodium pyruvate (Sigma) and penicillin 100 units/mL/streptomycin 0.1 mg/mL (Sigma), at 37°C in a humidified 5% CO_2_ atmosphere. Cells were cultured to 70% to 80% confluence with culture media being replaced every other day. The stimulus 2 μM sodium valproate (Sigma) was added directly to the existing media for 1 hour.

### 2.2 Total RNA preparation and cDNA synthesis

Total RNA was extracted from SH‐SY5Y cells using Trizol reagent (Invitrogen, Paisley, UK) and quantified using a NanoDrop 8000 Spectrophotometer (Thermo Fisher Scientific, Wilmington, DE, USA). Three micrograms of total RNA was reverse‐transcribed to single‐stranded cDNA using GoScript Reverse Transcription System (Promega, Southampton, UK) and random primers according to the manufacturers' instructions.

### 2.3 Polymerase chain reaction

Amplification of the MAOA uVNTR was performed using the primers previously described,^3^ forward 5′‐GAACGGACGCTCCATTCGGA‐3′ and reverse 5′‐ACAGCCTGACCGTGGAGAAG‐3′. Reactions contained 10 ng of DNA template, 5 pmol of each primer (Eurofins, Ebersberg, Germany), 1× GoTaq flexi buffer, 1 mM MgCl_2_ and 0.625 U of GoTaq DNA polymerase (Promega), 0.1 mM of each dNTP and 1 M betaine (Sigma). Cycling conditions were 2 minutes at 95°C initial denaturation, followed by 35 cycles of 20 seconds at 95°C, 20 seconds at 61°C and 30 seconds at 72°C and final elongation for 5 minutes at 72°C. Polymerase chain reaction (PCR) products were analysed using 1.5% agarose gels stained with ethidium bromide solution (Sigma) and visualised using a ultraviolet (UV) transilluminator.

### 2.4 Chromatin immunoprecipitation

Human SH‐SY5Y cells were grown to 80% confluence in a T175 flask and treated for 1 hour under the following conditions, basal (untreated), 2 μM sodium valproate and vehicle alone. Samples were processed using the methods described by Murgatroyd et al.^16^ The immunoprecipitations were performed using the following antibodies: anti‐Histone H3 (Abcam, Cambridge, UK), anti‐Nucleolin (Abcam), anti‐hnRNP K (Abcam), anti‐CCHC‐Type Zinc Finger Nucleic Acid Binding Protein gene (CNBP) (Abcam), anti‐RNA pol II CTD phospho Ser5 (Active Motif, Carlsbad, California), anti‐Sp1 and anti‐CCCTC‐Binding Factor gene (CTCF) (Millipore, Nottingham, UK). PCR analysis of the immunoprecipitated samples was performed using primers targeting the MAOA uVNTR as described above.

### 2.5 Methylation analysis

SH‐SY5Y genomic DNA (gDNA) was isolated and purified using QIAmp DNA Mini Kit (Qiagen, Manchester, UK). Genomic DNA was then fragmented by sonication using the Bioruptor Plus set at 10 cycles of 30‐second pulses followed by 30‐second pauses on high power. Three hundred nanograms of fragmented gDNA was incubated with CpG MethylQuest (Millipore) beads (Glutathione‐S‐Transferase ‐ Methyl Binding Domain (GST‐MBD) fusion protein) and binding buffer at room temperature for 1 hour with rotation. The supernatant was removed and stored for subsequent analysis, the beads were washed and the methylated DNA was eluted. Both methylated DNA (elutant) and unmethylated DNA (supernatant) were analysed by PCR of the MAOA uVNTR as previously described.

### 2.6 Saliva sample collection and RNA extraction

The saliva, was collected in the Oragene RNA container (DNA Genotek, ON, Canada) until the volume of saliva reached the indicated level marked on the collection tube, following the protocol outlined in the manufacturers' instructions. Five samples were obtained from children whose families were participating in the Wirral Child Health and Development Study (WCHADS). The WCHADS is funded by the Medical Research Council and run in partnership with National Health Service (NHS) Wirral, NHS Western Cheshire and Wirral University Teaching Hospital NHS Foundation Trust. It has received full ethical approval from Cheshire Local and North West 5 Research Ethics Committees (ref: 05/Q1506/107). Total RNA was extracted using Trizol reagent (Invitrogen) from the saliva samples. One microgram of total RNA was reverse‐transcribed to single‐stranded cDNA using GoScript Reverse Transcription System (Promega) and random primers, according to the manufacturers' instructions.

## 3 RESULTS

### 3.1 Human *MAOA* gene promoter architecture

The uVNTR was originally described as being 1.2 kb upstream of the TSS of the *MAOA* gene,^3^ however, later bioinformatic analysis from data in Hg19 and Hg38 of the human genome using ENCODE data on the USCS genome browser (http://genome.ucsc.edu)^17^ showed an additional TSS which is upstream of the uVNTR. Thus, the uVNTR can also be included in the 5′UTR of this MAOA isoform using the most 5′TSS, Figure 1. The ENCODE data shows those transcription factors determined by ChIP assay in a variety of cell lines to bind to this MAOA uVNTR/promoter region including CTCF and SP1 that bind Cytosine‐Guanine (CG)‐rich sequences in addition to RNA polymerase II (POLR2A). The sequence of this region of the MAOA promoter is rich in CpG given the 2 CpG islands overlapping both TSS.

### 3.2 Allelic‐specific regulation of MAOA expression

**Figure 2:**
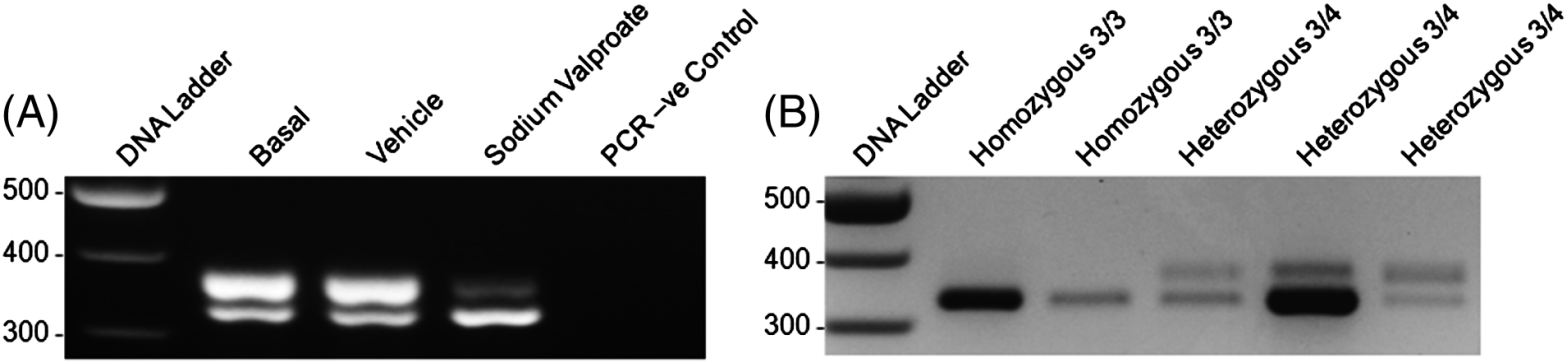
Expression of 2 MAOA isoforms: 1.5% agarose gel of MAOA uVNTR PCR amplification. Amplicon size 324 bp for 3R uVNTR, 354 bp for 4R uVNTR. A, SH‐SY5Y cell line cDNA: lanes from left to right: 100 bp ladder marker; basal condition; vehicle control: H_2_O 1 hour; sodium valproate treatment 1 hour; PCR negative control. B, Human female cDNA samples: lanes from left to right: 100 bp ladder marker; 2× homozygous 3R/3R uVNTR; 3× heterozygous 3R/4R uVNTR

We used the human neuroblastoma cell line SH‐SY5Y to address MAOA expression. SH‐SY5Y is a well‐characterised cell line which was originally isolated from a 4‐year‐old female. Genotyping of this cell line showed that it was heterozygous for the uVNTR with alleles containing either 3 or 4 copies of the repeat element. This allowed identification of allelic‐specific expression of the MAOA isoform which initiated from the most 5′ TSS because mRNA initiating here included the uVNTR in the 5′UTR. Under basal growth conditions we were able to detect MAOA mRNA corresponding to expression from both alleles using the uVNTR length as the distinguishing feature, Figure 2A. This PCR is the standard protocol extensively used and validated for addressing uVNTR variation at the level of DNA.^9^, ^11^ A variety of other PCRs across MAOA exons and control genes were utilised to confirm the fragment was not generated by contaminating genomic DNA in the cDNA (data not shown). Confirmation of the sequence of the MAOA fragments identified from the PCR was confirmed by sequence analysis of fragments after cloning into a reporter gene for future studies (data not shown). Similarly, analysis of cDNA prepared from saliva from females heterozygous for the uVNTR showed that in vivo expression from both alleles could be observed in the same individual, Figure 2B.

Attempts to use qPCR to measure allele‐specific expression over heterozygous VNTRs were problematic perhaps due to preferential amplification of a specific allele in early rounds of PCR and consistent, reproducible data was difficult to obtain. However, using the heterozygous cell line SH‐SY5Y we could utilise the size variation of the distinct uVNTRs to compare intensity between and within experiments for the same primer set; this showed that in SH‐SY5Y cells in response to exposure to sodium valproate, a mood stabiliser, a switch in the levels of MAOA allelic expression was observed. Specifically, the ratio of L‐ to S‐allele expression was reproducibly reversed, Figure 2A. This switch in relative levels of expression cannot be explained by PCR allele preference. As the *MAOA* gene is on the X chromosome the expression of both alleles in the SH‐SY5Y cells might require this region to escape X inactivation as has been previously reported for the MAOA locus.^12^, ^13^, ^14^ Alternatively, the expression of both alleles could be construed as a mosaic effect of random silencing of one allele in any particular cell and thus the population would appear to express both alleles.

**Figure 3:**
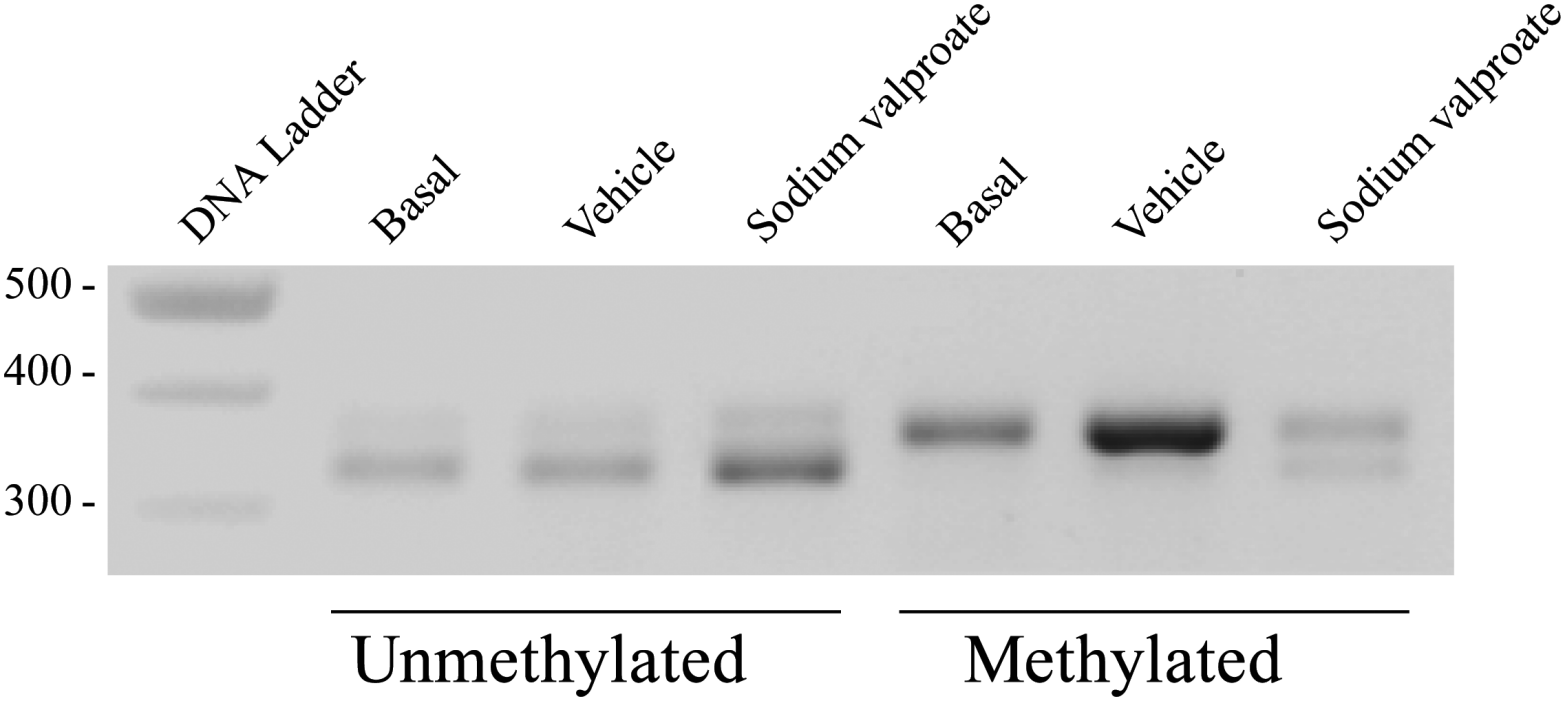
Methylation at the MAOA promoter. PCR amplification of sheared gDNA post CpG MethylQuest treatment. Amplicon size 324 bp for 3R uVNTR, 354 bp for 4R uVNTR. Lane 1, 100 bp ladder marker; lanes 2 to 4 PCR of uVNTR under basal condition; vehicle control: H_2_O; sodium valproate PCR of unmethylated DNA (not bound to methyl domain‐binding protein; CpG MethylQuest protein). Lanes 5 to 7, PCR of uVNTR under basal condition; vehicle control: H_2_O; sodium valproate treatment of methylated DNA (bound to methyl domain‐binding protein; CpG MethylQuest protein)

To explore the allelic expression further we analysed by ChIP transcriptional and epigenetic variation at the uVNTR domain in SH‐SY5Y cells in response to sodium valproate. The methylation status of both alleles was addressed by their ability to bind *Methyl*‐CpG‐*binding domain* (MBD). This showed, by addressing the ratio of each uVNTR allele, a clear specificity in that the 4 copy uVNTR promoter was predominantly methylated under basal growth conditions whereas the 3 copy uVNTR promoter was hypomethylated, Figure 3. The methylation pattern was altered when the cells were exposed to sodium valproate. Specifically, variation was mostly clearly observed in the methylated fractions, in which methylation could now be easily observed over the 3 copy allele; the ratio of methylated 4 copy allele to 3 copy allele was quite distinct from basal conditions, Figure 3. We therefore explored binding of RNA polymerase II and the transcription factors CTCF and SP1 identified from ENCODE data as binding to the uVNTR, Figure 1, to gain insight into potential transcriptional mechanisms operating at this locus.

**Figure 4:**
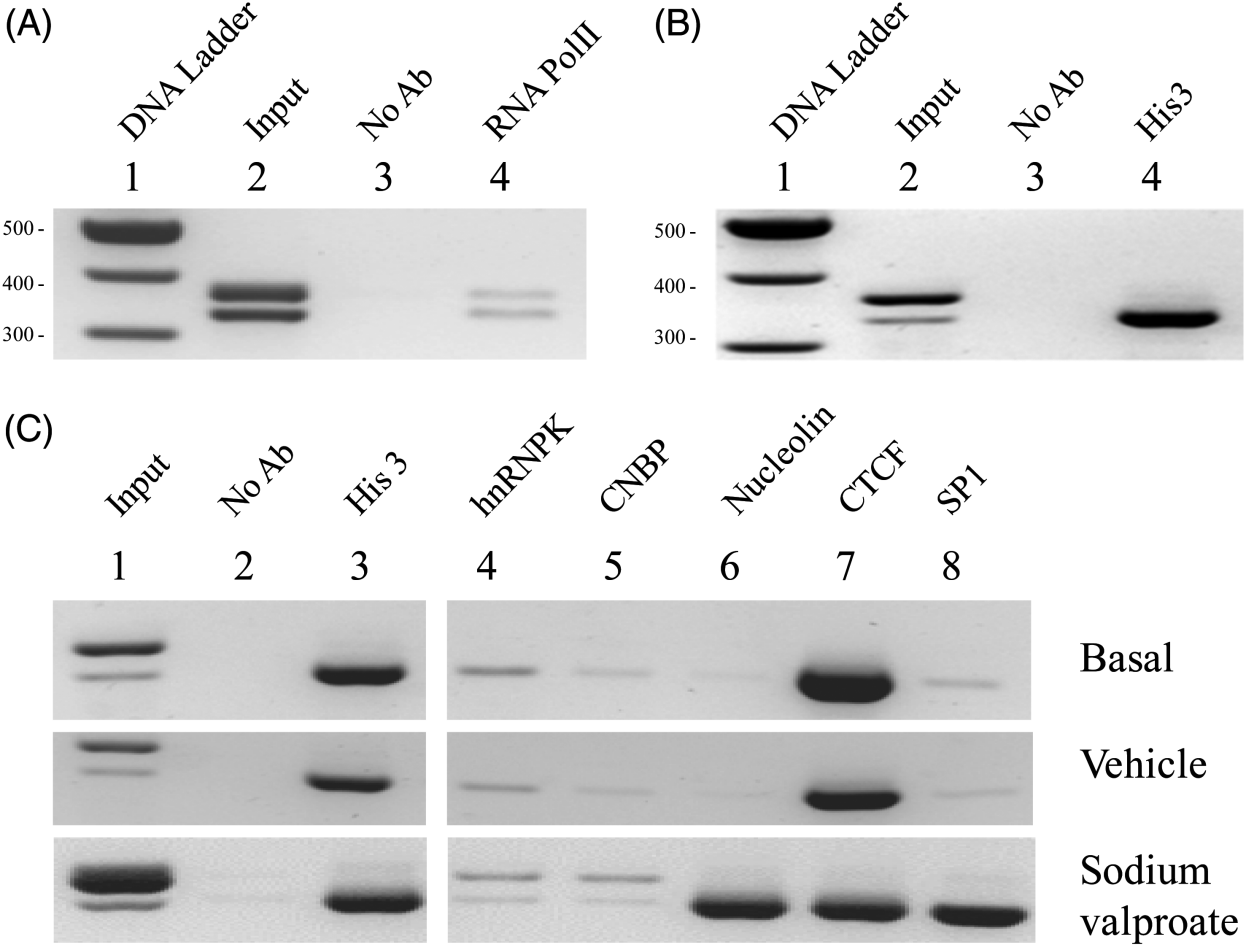
ChIP analysis of the MAOA VNTRs alleles. PCR amplification of sheared gDNA post‐enrichment by a specific antibody. Amplicon size 324 bp for 3R VNTR, 354 bp for 4R VNTR. A, in vivo interaction of active RNA pol II (CTD phospho Ser5) under basal growth conditions, lanes from left to right: 100 bp ladder marker; input DNA (1% sheared chromatin) acts as a positive control for PCR; no antibody: negative control for non‐specific background binding; active RNA pol II (CTD phospho Ser5) antibody. B, in vivo interaction of histone 3 with the MAOA uVNTR alleles under basal growth conditions. Lanes from left to right: 100 bp ladder marker; input DNA (1% sheared chromatin); no antibody; histone 3 antibody. C, ChIP analysis of the in vivo interaction of factors associated with MAOA uVNTR alleles. Under basal conditions (top panel), following 1 hour exposure to vehicle/H_2_O (middle panel), following 1 hour exposure to 2 uM sodium valproate (bottom panel). Lanes from left to right: lane 1 input DNA (1% sheared chromatin); lane 2 no antibody; lane 3 histone 3 antibody; lane 4 single stranded DNA (ssDNA) binding protein hnRNP K antibody; lane 5 ssDNA binding protein CNBP antibody; lane 6 G4 binding protein nucleolin antibody; lane 7 transcription factor CTCF antibody; lane 8 transcription factor Sp1 antibody

RNA polymerase II was showed to be present on both alleles under basal conditions, consistent with expression from both alleles, Figure 4A, lane 4. This was in stark contrast to Histone 3 (His3) under basal conditions, Figure 4B, lane 4 (His3), which was found predominantly on the shorter, 3 copy repeat, uVNTR allele. Similar to the observations obtained for His3, under basal growth conditions, or exposure to H_2_0 as the vehicle control, CTCF and SP1 transcription factor binding was predominantly observed on the short, 3 copy uVNTR allele, Figure 4C, top and middle panel, lanes 7 and 8. Upon exposure to sodium valproate, Figure 4C, bottom panel, lanes 8, there was a significant increase for SP1 binding to the short 3 copy allele which is correlated with the switch to increased expression of the longer 5′TSS containing the 3 copy uVNTR in the 5′UTR observed in Figure 2A.

The distinct promoter architecture is suggestive of a distinct/specific mechanism to regulate each MAOA‐allelic promoter based in part on uVNTR repeat copy number. In the literature, CTCF and SP1 both bind double stranded DNA but recognise the same sequences as the single stranded DNA‐binding proteins, hnRNPK and CNBP.^18^, ^19^ These single stranded DNA‐binding proteins were also bound predominantly to the short 3 copy allele under basal conditions, Figure 4C top and centre panel, lanes 4 and 5, but in response to valproate they now also bound the 4 copy allele, Figure 4C, bottom panel, lanes 4 and 5. This was in stark contrast to SP1 binding which was dramatically increased only on the 3 copy allele. In contrast the binding of CTCF was not significantly changed in response to valproate with any change in distribution, it remained bound predominantly to the 3 copy allele. CTCF function on transcription is varied, ranging from repression to transcriptional pausing and transactivation which in part is dependent on interacting partners, one of which is nucleolin which can mediate intra‐ and interchromosomal interactions.^20^ Analysis of nucleolin by ChIP indicated it binding weakly to the 3 copy allele under basal conditions with a vast increase in binding after exposure of the cells to valproate, Figure 4C, bottom panel, lane 6. It should be noted that nucleolin can also interact with SP1 and both these factors were dramatically increased in their binding to the short allele after exposure to valproate.

## 4 DISCUSSION

The MAOA uVNTR is one of the best characterised G × E interactions in the literature and the accepted hypothesis is that the different repeat copy numbers of the uVNTR direct the differential expression of MAOA in response to the environment. This is a relatively straightforward explanation for the male gender which has only one allele for MAOA. However, the situation is more complex in females for several reasons including (1) gene dosage, (2) the potential for MAOA to escape X‐inactivation and (3) heterozygosity of the uVNTR whose genotype might direct distinct *MAOA* gene expression patterns.

We investigated the molecular mechanisms underlying MAOA expression in the human neuroblastoma cell line SH‐SY5Y which is both female and heterozygous for the uVNTR. This heterozygosity of the uVNTR was crucial for our study to allow us to correlate mRNA expression from each MAOA allele with transcription factor binding and epigenetic marks over the uVNTR. Our data indicated that both alleles of the *MAOA* gene were active in SH‐SY5Y cells, albeit by very distinct molecular mechanisms based on the binding of proteins and methylation marks over this region. Most noticeably although both alleles had very different chromatin structures (methylation and transcription factor interactions), when ChIP was performed for an active RNA polymerase, binding was observed at both alleles which was consistent with the cDNA studies showing that both alleles were active despite the distinct chromatin architecture, Figure 4A. The methylation of one allele which apparently did not repress MAOA expression, Figure 3, would be consistent with recent work on X chromosome gene expression patterns which indicated that methylation did not necessarily correlate with gene repression but rather functioned as a parameter affecting a region's ability to escape X inactivation.^13^ To further investigate the regulation of *MAOA* gene expression by the uVNTR we analysed binding for 5 transcription factors: CTCF, SP1, hnRNP K, CNBP and nucleolin. Our data provided evidence that these proteins recognised the proximal MAOA promoter encompassing the uVNTR and more importantly a distinct pattern of binding was observed over each individual allele which was modulated both by genotype of the uVNTR and exposure of the cells to sodium valproate, Figure 4C. This data was consistent with a role for gender in regulation of MAOA expression via epigenetic mechanisms, for example, the gender‐specific changes of methylation in a longitudinal study of twins^21^ and hypomethylation in the pathogenesis of panic disorder particularly in female patients.^22^

Our model could in part explain the differences in uVNTR genotype association for specific mental health issues when male and females are compared, such examples include: MAOA uVNTR moderating the relationship between childhood maltreatment and dysthymia only in females,^23^ gender differences including pronounced effects on Attention‐deficit hyperactivity disorder (ADHD) and anxiety in females whereas the opposite is true for autism, bipolar disorder and aggressive behaviour in males.^4^, ^24^ Indeed many studies of MAOA G × E report only on males due to compounding affects including female heterozygosity for the uVNTR.^6^, ^8^, ^11^ This regulation could be further complicated in that the *MAOA* gene is one of the approximately 15% of X chromosome genes estimated to escape X inactivation.^12^, ^13^, ^14^ However, there is still much debate about X inactivation in vivo and there may be no reason why X‐inactivation of the second allele in females itself is not dependent on G × E interactions. Consistent with this, the gene termed a master regulator of X chromosome inactivation, X Inactive Specific Transcript gene (XIST), is over expressed in females with major affective disorders.^25^ Thus, it is likely that both sex‐based and disorder‐based differences exist and our study highlights one potential mechanism to explain such differences.
